# A Case of Hyper IgD and Periodic Fever Syndrome in Japan

**DOI:** 10.4137/ccrep.s722

**Published:** 2008-05-16

**Authors:** Hisashi Kawashima, Akiyoshi Hoshi, Hiroaki Ioi, Chiako Ishii, Satoshi Sato, Yasuyo Kashiwagi, Kouji Takekuma, Akinori Hoshika

**Affiliations:** Department of Paediatrics, Tokyo Medical University.

**Keywords:** IgD, periodic fever, mevalonate kinase, PFAPA

## Abstract

We report a four-year-old Japanese girl with hyper IgD and periodic fever syndrome. There is a first report of hyper IgD syndrome (HIDS) of which the genomic study was done in Japan. In this report a girl complained of periodic fever and abdominal symptoms accompanied with high levels of mevalonic acid in urine and serum. She has been well controlled by non-steroidal anti-inflammatory drugs (NSAIDs) for 3 years.

## Introduction

There is a first report of hyper IgD syndrome (HIDS) of which the genomic study was done in Japan. In this report a girl complained of periodic fever and abdominal symptoms accompanied with high levels of mevalonic acid in urine and serum. We investigated the genomic assay, and the common mutations were negative. She has been well controlled by non-steroidal anti-inflammatory drugs (NSAIDs) for 3 years.

## Case Report

A four-year-old girl was admitted because of fever and vomiting. Her family and past histories were unremarkable except for bronchial asthma of her father. She had frequent and periodic fever since she was two years old. She had been admitted five times because of prolonged fever, and diagnosed as pneumonia, tonsillitis, acute otitis media and sinusitis. She had febrile periods (38 °–40 °C) lasting 4 to 7 days every 4 to 8 weeks. She often complained of mild arthralgia in knee joints and had diarrhea frequently. She often showed cervical lymphoadenopathy, tonsillitis and hepatomegaly without stomatitis. She was admitted at this time because of fever which continued for 5 days, vomiting and increased acute inflammatory laboratory data (white blood cell 11.000/μl, CRP 8.9 mg/dl). She also complained of sore throat and diarrhea.

**Figure 1:**
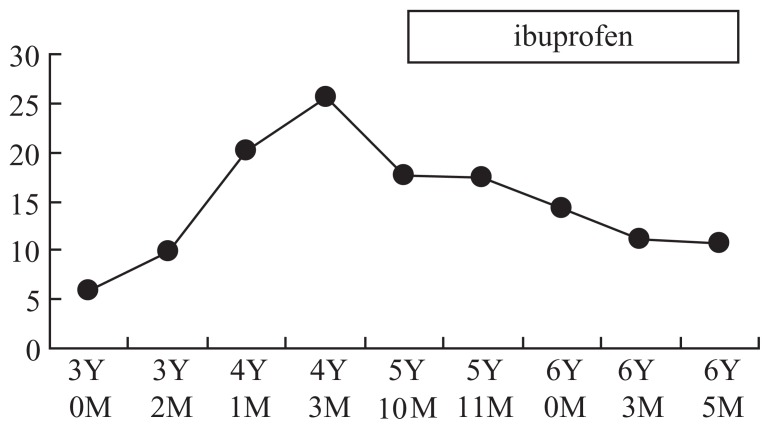
Fluctuation of serum IgD (mg/dl).

Her height was 104 cm (+0.93SD), and weight was 14 kg (−0.66SD). Her blood pressure was 114/72 mmHg, and heart rate was 128 per minutes, respiratory rate was 26 per minutes. Her tonsil was reddish and enlarged. Cervical lymphoadnopahty was accompanied without hepatosplenomegaly. Her laboratory findings were not remarkable except for increased acute inflammatory responses. All bacterial cultures were negative. Her urinalysis was normal. Chest and sinusoidal X-ray, abdomen and heart echogram were all normal. Without antibiotics her fever decreased naturally after 8 days, and her CRP and count of White blood cells normalized. Her IgD was extremely high as 25.69 mg/dl (181.9 IU/ml). Other laboratory findings were as follows: white blood cells 11.000/μl (neutrophiles 78.9%, eosininophile 0.0%, basophile 0.3%, lymphocytes 13.9%, monocytes 6.9%), red blood cells 4.15 × 10^6^/μl, Hemoglobin 11.0 g/dl, hematocrit 33.8%, platelets 196 × 10^3^/μl, total protein 7.3 g/dl, albumin 4.1 g/dl, aspartate transaminase (AST) 28 U/L, alanine aminotransferase (ALT) 3 U/L, Lactate Dehydrogenase (LDH) 521 U/L, alkaline phosphatase 278 U/L, γ-glutamyltranspeptidase (GTP) 10 U/L, Total birilubin 0.51 mg/dl, BUN 8.7 mg/dl, Creatinin 0.22 mg/dl, beta-2 microglobulin 1.14 mg/l, ferritin 126.1 ng/dl, CRP 8.9 mg/dl, Erythrocyte Sedimentation Rate 91 mm/hour, 2–5 oligoadenylate synthetase 180 pmol/dl, IL-6 15 pg/ml, Serum Amyloid A (SAA) 1100 μg/l (<8 μg/l), IgG 1488.0 mg/dl, IgA 127.0 mg/dl, IgM 180.0 mg/dl, IgE 848.1 IU/ml. IgG subclass were within normal range. RAST was positive for egg white. C3, C4 and CH50 were 167 mg/dl, 44 mg/dl, 52.3 U/ml, respectively. Anti-nuclear antibody, anti-DNA antibody and LE factor were negative. ASLO was below 60 IU/ml. The titer for adenovirus (CF) was below ×4. EBV-VCA IgM and EBV-EBNA were below ×10. EB virus DNA was below 2.0 × 10/10^6^cells. Mevalonic acid showed 10 ng/ml in serum, and 115 ng/ml in urine, respectively. Both levels were higher than those of healthy controls. Restriction Fragment Length Polymorphism (RFLP) and direct sequencing for V377I and I268T were negative [1]. From the data of high IgD and her clinical symptoms she was diagnosed as non-classic HIDS. Without tonsillectomy ibuprofen (20 mg/kg) was effective to shorten the duration of febrile episodes (from 7 to 2–3 days) for 3 years. However, the levels of IgD were constantly high without any other symptoms. The levels of IgD were normal before 4 years earlier, which are shown in Figure 1.

## Discussion

This patient showed periodic fever since she was 2 years old, accompanied with high IgD levels and other clinical symptoms (diarrhea, vomiting and arthralgia) which coincided with HIDS. From these data Periodic fever with Aphtous Pharyngitis Adenitis (PFAPA), chronic EB virus infection, Familial Mediterranean fever (FMF), TNF receptor-associated periodic syndrome (TRAPS), and Bechet disease were also suspected. Chronic EV virus infection was not likely because of a lack of EB virus DNA. Symptoms of FMF usually appear at later age, and those of TRAPS appear at an earlier age. From the symptoms of this patient it was difficult to diagnose with PFAPA. Unlike PFAPA, abnormal symptoms, especially vomiting (56%) and diarrhea (82%), are dominant features, and 80% have polyarthralgia; aphthous stomatitis was not a manifestation in HIDS [2]. Her IgD levels were constantly high and mevalonic acid in urine and serum showed high. The data coincide with that of HIDS. Yoshimura et al. reported a boy having periodic fever since 3 years and 2 months [3]. Okamoto reported an other case aged 4 years who showed periodic fever and ectodermal dysplasia [4]. None of the reports mentioned about the concentration of mevalonic acid and genomic studies of mevalonate kinase. We analyzed the mevalonate kinase (MVK) gene of variation V377I and I268T. HIDS is divided into two types; classic and non-classic. The classic type is defined to show the mutation of the MVK gene. The gene variation of MVK which are known, are V377I/I268T/P167L/H20P/A344T as the main amino acid variation of the MVK. V377I is the most frequent mutation with amino acid change [1, 5]. According to the study of Cuisset et al. they found 20 out of 25 unrelated cases showing V377I [5]. Since the patient revealed high mevalonic acid, she might have other heterozygote compound except for V377I. A more comprehensive molecular screening including TNFRSF1A is needed [6]. The assay of activity of mevalonic acid kinase would help to diagnose non-classic form of HIDS.

Several trials of therapies for HIDS have been done. Simvastatin, thalidomide, etanercept and anakinra have been tried with good response [7,8,9]. However, most therapies effect the course of periodic fever temporally. Picco et al. also reported the effect of NSAIDs in the treatment of hyper-IgD syndrome [10]. On the other hand there is only one report of the non-effectiveness of NSAIDs [11]. In our case NSAIDs was effective to control her symptoms especially the duration of febrile episodes and arthralgia.
